# Intra-Abdominal Effusions of Traumatic Origin

**Published:** 1919-01

**Authors:** 


***Importance of the Site and Diagnostic Value of Dull Areas in the Estimation of Intra-Abdominal Effusions of Traumatic Origin.*** By CONSTANTINI and Dr. VIGOT. *Paris Médical*, November 2, 1918.

The intraperitoneal effusions occurring in cases of deep, abdominal wounds, or contusions complicated by visceral or vascular lesions, are serious when accompanied by hemorrhage and require immediate surgical action.

As hemorrhage always implies sufficient effusion to produce dull areas, the importance of ascertaining the latter is obvious. Out of the three symptoms which show the existence of visceral lesions — abdominal contracture, pre-hepatic sonority and iliac dullness — only the third has been proved to be fully reliable. In the research for this sign, it must be borne in mind that, in accordance with the law of gravity, the position of the liquid contained in the abdomen varies with that of the patient's body. If, for example, the patient is in a reclining position, all the liquid runs into the Douglas culdesac; if he is lying on the right or left side, the liquid accumulates in that side.

Two positions have been considered :

(1) The patient is in a reclining position — the liquid falls into the Douglas pouch.

The liquid falls into the anatomically free iliac pit, which, in most cases, is the right one, as can be easily seen from an anatomic study of the abdomen. In some cases, however, the coecum is placed very low down and close to the Fallopian arch. On the left, the meso-sigmoid is abnormally short and the liquid, running under it instead of above, comes into contact with the abdominal wall, thus producing a dull area on the left and exaggerated sonority on the right.

Iliac dullness is determined by comparing the sonority of the 2 iliac pits. Intervention should always follow in the case of a clearly ascertained difference.

(2) The patient is lying on the side.

In this case, a dull area is found on the side on which the patient has been lying; the sonority of both pits remains normal.

